# Development and Assessment of a Novel Core Biopsy-Based Prediction Model for Pathological Complete Response to Neoadjuvant Chemotherapy in Women with Breast Cancer

**DOI:** 10.3390/ijerph20021617

**Published:** 2023-01-16

**Authors:** Ailin Lan, Junru Chen, Chao Li, Yudi Jin, Yinan Wu, Yuran Dai, Linshan Jiang, Han Li, Yang Peng, Shengchun Liu

**Affiliations:** 1 Department of Breast and Thyroid Surgery, The First Affiliated Hospital of Chongqing Medical University, No. 1 Youyi Road, Yuzhong District, Chongqing 400016, China; 2 Department of Cardiothoracic Surgery, The First Affiliated Hospital of Chongqing Medical University, No. 1 Youyi Road, Yuzhong District, Chongqing 400016, China; 3 Department of Vascular Surgery, Southwest Hospital, Army Medical University, 38 Main Street, Gaotanyan, Shapingba, Chongqing 400038, China; 4 Department of Pathology, Chongqing University Cancer Hospital, No. 181, Hanyu Road, Shapingba District, Chongqing 400030, China

**Keywords:** breast cancer, neoadjuvant chemotherapy, pathological complete response, prediction model, nomogram

## Abstract

Purpose: Pathological complete response (pCR), the goal of NAC, is considered a surrogate for favorable outcomes in breast cancer (BC) patients administrated neoadjuvant chemotherapy (NAC). This study aimed to develop and assess a novel nomogram model for predicting the probability of pCR based on the core biopsy. Methods: This was a retrospective study involving 920 BC patients administered NAC between January 2012 and December 2018. The patients were divided into a primary cohort (769 patients from January 2012 to December 2017) and a validation cohort (151 patients from January 2017 to December 2018). After converting continuous variables to categorical variables, variables entering the model were sequentially identified via univariate analysis, a multicollinearity test, and binary logistic regression analysis, and then, a nomogram model was developed. The performance of the model was assessed concerning its discrimination, accuracy, and clinical utility. Results: The optimal predictive threshold for estrogen receptor (ER), Ki67, and p53 were 22.5%, 32.5%, and 37.5%, respectively (all *p* < 0.001). Five variables were selected to develop the model: clinical T staging (cT), clinical nodal (cN) status, ER status, Ki67 status, and p53 status (all *p* ≤ 0.001). The nomogram showed good discrimination with the area under the curve (AUC) of 0.804 and 0.774 for the primary and validation cohorts, respectively, and good calibration. Decision curve analysis (DCA) showed that the model had practical clinical value. Conclusions: This study constructed a novel nomogram model based on cT, cN, ER status, Ki67 status, and p53 status, which could be applied to personalize the prediction of pCR in BC patients treated with NAC.

## 1. Introduction

According to the latest global cancer data, breast cancer (BC) is the most commonly diagnosed female malignant tumor and the fifth leading cause of cancer mortality [1]. Core biopsy, regarded as the gold standard for tissue acquisition for pathological assessment when considering malignancy, is widely used in BC patients. According to core biopsy, biomarkers including estrogen receptor (ER), progesterone receptor (PR), Ki67, p53 protein, and human epidermal growth factor receptor 2 (HER2) expression could be measured via immunohistochemistry (IHC) or fluorescence in situ hybridization (FISH). Breast cancers are divided into several subtypes according to ER, PR, Ki67, and HER2. These subtypes differ in tumor biological behavior and pathological characteristics, and statistically significant differences in the pathological responses between the different subtypes have been previously reported in the literature [2]. Additionally, the immunohistochemistry biomarker p53 is frequently used in the pathological analysis of BC. p53 signaling system anomalies are seen in almost all human tumors, and p53 mutations are found in over 50% of malignant tumors [3] and 30% of breast cancers [4], which is the main driving force behind p53 being a hot topic. In contrast to hormone receptors (HRs) and HER2, the significance of p53 in breast cancer is frequently disregarded. This is likely because no p53-based treatments have been licensed yet. The notion that there is no treatment for p53 is increasingly being disproved, according to a high-impact review that was just released [5]. Based on the centrality of p53 in human cancer, it has the potential to revolutionize cancer treatment if successful. Although p53 has not been included in international guidelines for the time being for the reasons mentioned above, there is a large consensus that studies on the efficacy of neoadjuvant chemotherapy (NAC) in BC have shown significantly higher pathological complete response (pCR) rates in tumors with p53 mutations compared to wild-type tumors [6]. Molecular profiling is routinely applied to pre-treatment core biopsy to assist in pre-operative management decisions, which is a vital reference when deciding whether to proceed with NAC treatment [7].

NAC, an effective pre-operation treatment, is widely applied in patients with locally advanced BC or the molecular subtype of tumors associated with a high likelihood of response [8]. The primary aim of NAC is to downstage tumor staging and convert inoperable tumors to resectable ones [9]. Furthermore, it may provide crucial prognostic information based on the response to NAC [10]. Other advantages of NAC, according to the Breast Cancer NCCN Guidelines Version 3.2022, include giving patients who are having mastectomy surgery time to arrange breast reconstruction and undergo proper genetic testing [11]. It may offer a chance to find patients who are candidates for clinical trials of innovative medicines in the adjuvant context for those with considerable residual illness after conventional NAC [11].

pCR has been considered a reliable predictive factor, since achieving pCR is related to favorable survival outcomes irrespective of BC molecular subtypes in patients who received NAC [12]. However, due to the heterogeneity of BC, patients’ responses to NAC are differential, which makes it possible for patients with analogous clinical TNM staging to have diverse outcomes [13]. Recent studies have concentrated on individualized therapy by increasing and de-escalating treatment, respectively, for non-responders and great responders [14,15]. The ability to predict pCR before NAC is helpful for modifying therapy plans and choosing patients for clinical studies on de-escalating treatments. In addition, knowing the pCR prior to surgery makes it easier to modify the optimal surgical plan, especially for axillary management [16]. Therefore, discrimination against these heterogeneities and the accurate prediction of responses to NAC based on data available in the early pre-treatment period is meaningful; it may inform the therapeutic options, thereby impacting the treatment selection.

A vital unresolved clinical issue to consider when selecting NAC is the possibility of future pCR. Today, some predictors of response to NAC have been reported in the previous literature, including clinical, pathological, radiographic, and biological parameters [17,18,19,20]. Clinical information based on TNM staging at the time of the diagnosis of cancer patients provides the basis for the implementation of NAC and provides a possible prognosis based on the average survival of comparable groups with similar levels of disease [17]. These factors, such as lymph node status [21], tumor size [22], and immunohistochemical markers [23], are associated with pCR. In addition, the pCR of NAC can be predicted from different perspectives, including whole-slide biopsy images [24], genomic features [25], and even radiological features from pre- [26] and mid-treatment magnetic resonance imaging (MRI) [27]. Based on these parameters, quite a few prediction models have been built [28,29]; however, the prognostic biomarkers involved in most models are not accessible to every patient for economic reasons or due to the cumbersome steps involved. These reasons limited the assessment of which of the NAC-treated patients would have a good response. To fill this research gap, we developed and assessed a novel prediction model based on the core biopsy at the time of diagnosis. In our setting, the well-established predictive factors incorporate clinical T staging (cT), clinical nodal (cN) status, ER status, Ki67 status, and p53 status. These factors, identified as prognostic factors, were discussed in previous studies [6,30,31]. To improve the accuracy of the prediction model, we found the predictive cut-off value of pre-treatment ER, Ki67, and p53 and combined them with other clinical predictive factors. To our knowledge, this is the first pCR prediction model in BC patients who received NAC treatment that involves the expression level of pre-treatment p53 protein with a specific value. This novel model may provide a convenient and economical method to predict the response to NAC.

## 2. Patients and Methods

### 2.1. Study Subjects

**Figure 1:**
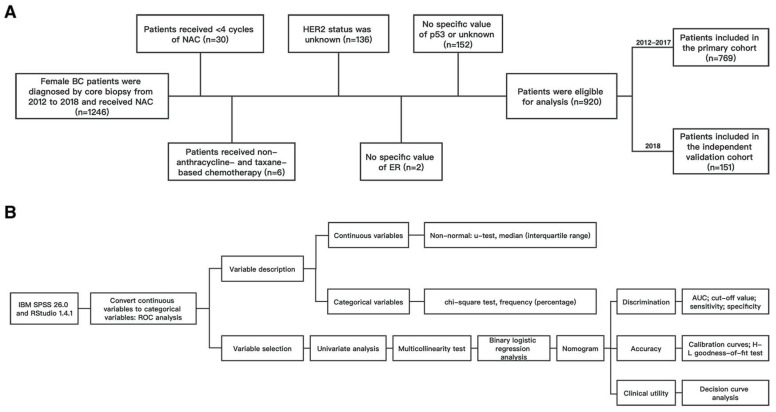
Flow chart of (**A**) patient selection; (**B**) statistical process. Abbreviations: BC, breast cancer; NAC, neoadjuvant chemotherapy; HER2, human epidermal growth factor receptor 2; ER, estrogen receptor; ROC, receiver operating characteristic; AUC, area under the curve.

This was a retrospective analysis of female patients with core-biopsy-confirmed invasive BC at our institution between January 2012 and December 2018. This study was approved by the ethics committee of First Affiliated Hospital of Chongqing Medical University (ID: No. 2020–59) and according to the Declaration of Helsinki; as a result of a retrospective observational study design, the hospital waived the request for informed consent. Female patients ≥ 18 years old who underwent NAC and subsequent surgery were incorporated into this study. Patients were excluded if (1) they received < 4 cycles of NAC; (2) they received non-anthracycline- and taxane-based chemotherapy; (3) their HER2 status was unknown; (4) no specific values of ER, PR, Ki67, or p53 were available or unknown on core biopsy. Finally, a total of 920 eligible patients were included in this study (Figure 1A). According to the time of diagnosis, patients were divided into a primary cohort (769 patients between January 2012 and December 2017) and a validation cohort (151 patients between January 2017 and December 2018).

### 2.2. Pathologic Assessment

All specimens from core biopsies were fixed with formalin solution within 2 h of isolation and then delivered to the department of pathology, Chongqing Medical University. All were processed in the pathology department by the same standards according to immunohistochemistry-related procedures. Immunohistochemical analysis was carried out on an immunohistochemical autostainer (Leica Bond-Max, Milton Keynes, UK) following a tested and optimized protocol of immunohistochemistry. Additionally, the following ready-to-use antibodies were used: ER (clone 1D5), PR (clone PgR636), HER2 (clone 4B5), Ki67 (clone MIB-1), and p53 (clone DO-7). The interpretation of immunohistochemical results referred to the following steps: 5 high-power fields were randomly observed in the “hottest spot” area of the tumor, and 100 tumor cells were assessed per field. Tumor cells with strong nuclear immunostaining were defined as positive cells, and then, the average percentages (range 0–100%) of positive cells of the five fields for each molecular maker (ER, PR, Ki67, and p53) were calculated. Two expert pathologists evaluated this procedure independently, and if there was a 10% or less discrepancy in the results of the two observers’ counts, the observations were deemed to be consistent. If not, the data were re-evaluated (unblinded), and a consensus was obtained. The outcome of the immunohistochemical interpretation was determined by taking the average of the positive percentages determined by two observers. The Sauter et al. [32] standards were used to assess the HER2 status as 0, 1+, 2+, or 3+. According to the 2011 St. Gallen consensus [33], scores of 0 and 1+ were negative, 2+ were ambiguous, and 3+ were positive. HER2 gene copy levels were assessed using FISH on tumors that had a 2+ staining. The absolute HER2 gene copy numbers and the ratio of HER2 gene copy numbers to corresponding chromosome 17 centromere (CEP17) numbers were obtained. The amplification of HER2 was defined as a HER2/CEP17 ratio ≥ 2.2. The nuclear staining of ER/PR via IHC with <1% positive tumor cells was characterized as ER/PR-negative, whereas the nuclear staining with ≥1% positive tumor cells was defined as ER/PR-positive, following ASCO/CAP guidelines [34]. ER and/or PR positivity is referred to as HR positivity. Based on the receptor status, the primary tumors were classified into four subtypes: HR-positive/HER2-negative, HR-positive/HER2-positive, HR-negative/HER2-positive, and HR-negative/HER2-negative. The pathological response of tumors to NAC was assessed by the Miller–Payne scoring system [35]. pCR was regarded as ypT0/Tis ypN0, which means no residual malignant cells in any excision of breast tissues and lymph nodes after the completion of NAC treatment [36].

### 2.3. Clinical Assessment

The clinical nodal status was assessed via clinical diagnostic imaging (ultrasonography and MRI).

### 2.4. Data Processing and Analysis

IBM SPSS 26.0 software (version 26.0, IBM Statistics, Chicago, IL, USA) was adopted to process data. In the primary cohort, receiver operating characteristic (ROC) analysis was performed to measure the cut-off value of ER, PR, Ki67, and p53 indications to convert continuous variables to categorical variables. The Kolmogorov–Smirnov goodness-of-fit test was utilized to assess the normality of continuous variables. Those that conformed to an abnormal distribution used the Mann–Whitney U test, described as a median and interquartile range. Categorical variables were described as frequencies and percentages and assessed using the chi-square test. In the primary cohort, independent variables were included in the univariate analysis; those variables with *p* < 0.05 were involved in the multicollinearity test to examine whether there was multicollinearity amongst the variables. A variance inflation factor (VIF) > 5 was regarded as strong collinearity. The selected variables were added to the binary logistic regression analysis with a forward LR method to obtain the independent predictive factors for pCR in the NAC setting.

### 2.5. Nomogram Development and Assessment

The RStudio 1.4.1 software and the IBM SPSS 26.0 software were used to develop and assess the model. The variables selected in the binary logistic regression model of the primary cohort were used as the final predictors to construct a nomogram predicting the likelihood of pCR using the “rms” extension package. We assessed the model in terms of the following aspects: discrimination, accuracy, and clinical utility. Internal and external validations of our prediction models were evaluated in both the primary and validation cohorts. The internal validation of the nomogram model was carried out using the Bootstrap method. Calibration curves were plotted using the “rms” extension package. The area under the curve (AUC) was used to assess the discriminative power of this nomogram model; in addition, the sensitivity and specificity of the model cut-off values were calculated. The Hosmer–Leme show test was utilized to evaluate the consistency of the model; *p* > 0.05 showed that the model prediction was in good agreement. Decision curve analysis (DCA) was utilized to judge the models’ clinical utility and predictive value. Considering it was the first pCR prediction model in BC involving the expression level of the pre-treatment p53 protein with a specific value, DCAs of the model with p53 and the model without p53 were performed. DCA curves and clinical impact curves were plotted using the “rmda” extension package. The flow chart of the statistical process is shown in Figure 1B.

## 3. Results

### 3.1. Convert Continuous Variables to Categorical Variables

**Figure 2:**
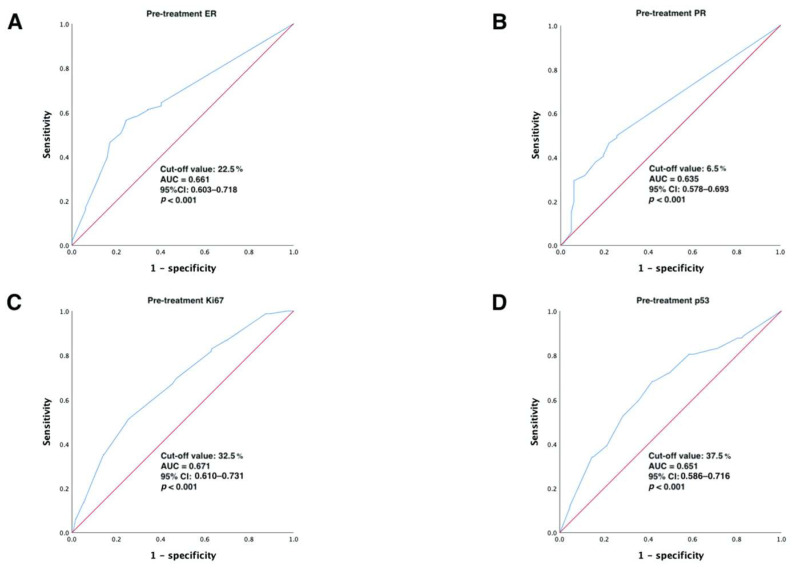
ROC analysis showing the best discriminative value to predict pCR for (**A**) ER; (**B**) PR; (**C**) Ki67; (**D**) p53 in the primary cohort. Abbreviations: ROC, receiver operating characteristic; AUC, area under the curve; CI, confidence intervals; ER, estrogen receptor; PR, progesterone receptor.

ROC curve analysis was utilized in the primary cohort to calculate the optimal cut-off value for pre-treatment ER, PR, Ki67, and p53 indications to convert continuous variables to categorical variables. Our results showed that the optimal cut-off values for ER, PR, Ki67, and p53 were 22.5% (95% CI: 0.603–0.718), 6.5% (95% CI: 0.578–0.693), 32.5% (95% CI: 0.610–0.731), and 37.5% (95% CI: 0.586–0.716), respectively (all *p* < 0.001), as detailed in Figure 2. Then, the continuous variables were transformed into categorical variables based on their cut-off values.

### 3.2. Patient Baseline Characteristics in the Primary Cohort and Univariate Analysis

In total, 769 eligible subjects in the primary cohort were enrolled in the analysis. A total of 82 patients (10.7%, 82/769) achieved pCR, while 687 patients (89.3%, 687/769) did not achieve it. The univariate analysis of predictive factors between the pCR group and non-pCR group showed statistically significant distinctions (*p* < 0.05) in terms of the six factors of cT, cN, ER status, PR status, Ki67 status, and p53 status, while there were no statistically significant distinctions between the two groups between age at diagnosis, menopausal status, HER2 status, and chemotherapy cycles. Details are shown in Table 1. Then, the six statistically significant predictors derived above were used to conduct a multicollinearity test. Since the VIFs of these predictors were all < 5, there was no covariance. Then, the above predictive factors were assigned values for binary logistic regression analysis, and the results of the multicollinearity test and assignments are summarized in Table 2.

### 3.3. Binary Logistic Regression Analysis

The binary logistic regression analysis was conducted with the six statistically significant predictive factors derived from the primary cohort described above (cT, cN, ER status, PR status, Ki67 status, and p53 status) as the independent variables and the achievement of pCR (“no” = 0, “yes” = 1) as the dependent variable. Finally, cT, cN, ER status, Ki67 status, and p53 status (all *p* ≤ 0.001) were independent predictors of pCR; these results are detailed in Table 3.

### 3.4. Develop and Assess the Nomogram

**Figure 3:**
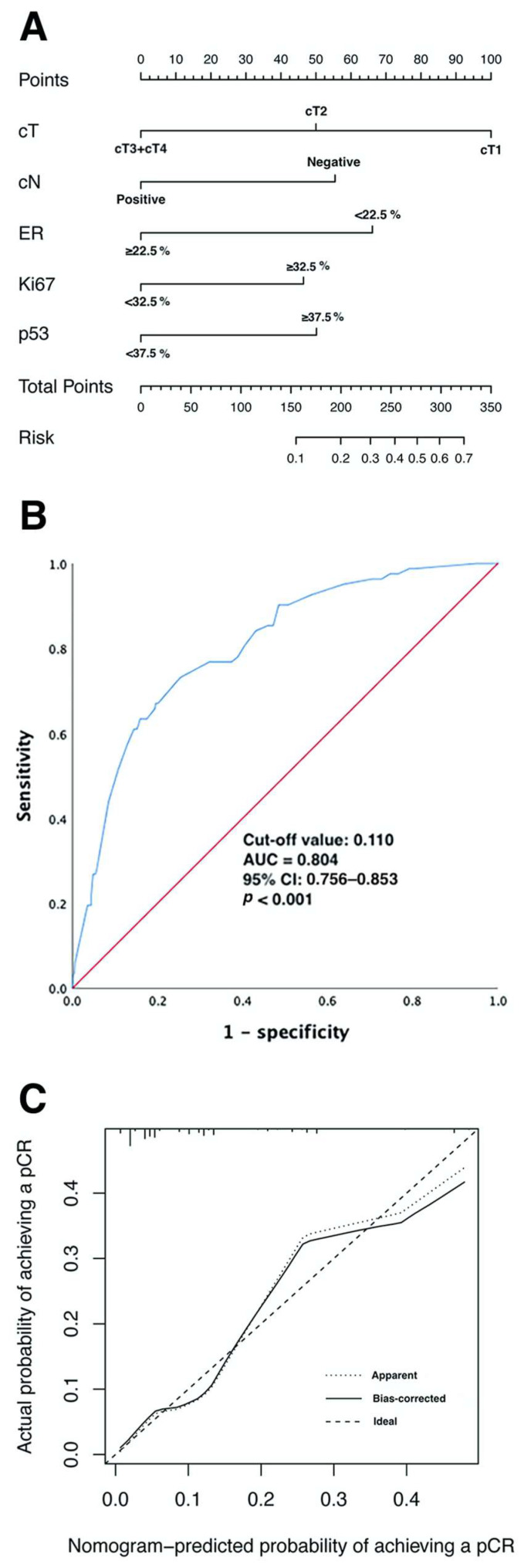
(**A**) Predicting the probability of pCR in the nomogram. (**B**) ROC curves for the nomogram model to predict the probability of achieving pCR in the primary cohort. (**C**) Calibration curves for the nomogram model predicting the probability of achieving pCR in the primary cohort. Abbreviations: pCR, pathological complete response; cT, clinical T staging; cN, clinical nodal status; ER, estrogen receptor; ROC, receiver operating characteristic; AUC, area under the curve; CI, confidence intervals.

We constructed the nomogram shown in Figure 3A based on the predictive factors derived from the binary logistic regression analysis of the primary cohort described above. The scores corresponding to five indicators (cT, cN, ER status, Ki67 status, and p53 status) of each patient in the nomogram were summed to calculate the final score, and the probability of achieving pCR for that patient could be derived.

As shown in Figure 3B, the ROC curve was utilized to evaluate the discrimination of the nomogram model in the primary cohort. The ROC analysis showed AUC = 0.804 (95% CI: 0.756–0.853; *p* < 0.001), the cut-off value was 0.110, and the sensitivity and specificity of the value were 73.2% and 74.7%, respectively.

The internal validation of the nomogram model was conducted using the Bootstrap method, and the calibration curve was plotted after 1000 replicate samples of the data from the primary cohort (Figure 3C). The Hosmer–Lemeshow goodness-of-fit test showed χ^2^ = 7.089, *p* = 0.527, suggesting that the model had good prediction accuracy.

**Figure 4:**
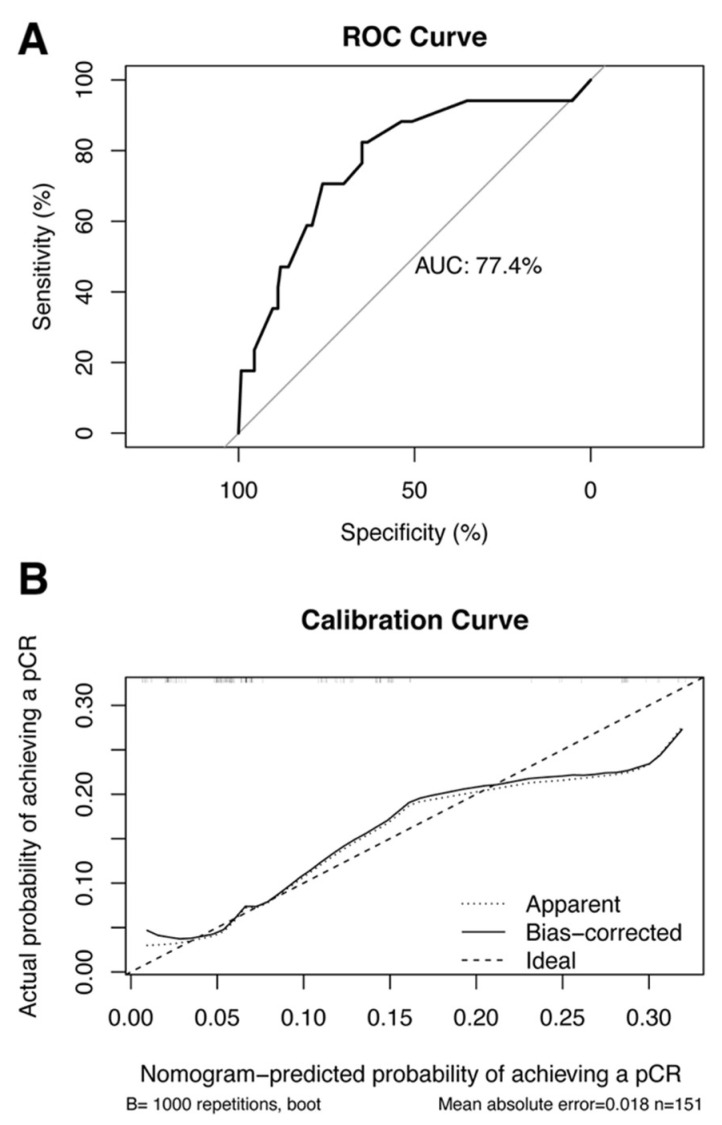
(**A**) ROC curves for the nomogram model to predict the probability of achieving pCR in the validation cohort. (**B**) Calibration curves for the nomogram model predicting the probability of achieving pCR in the validation cohort. Abbreviations: ROC, receiver operating characteristic; pCR pathological complete response; AUC, area under the curve.

We also performed external validation, and the clinical characteristics of the primary and validation cohorts are shown in Supplementary Table S1. The model was well discriminated, with an AUC of 0.774 (95% CI: 0.649–0.899) for the validation cohort (Figure 4A). In addition, the calibration plots showed good agreement in the validation cohort (Figure 4B). The relationship between cancer subtypes and pCR is shown in Supplementary Table S2.

**Figure 5:**
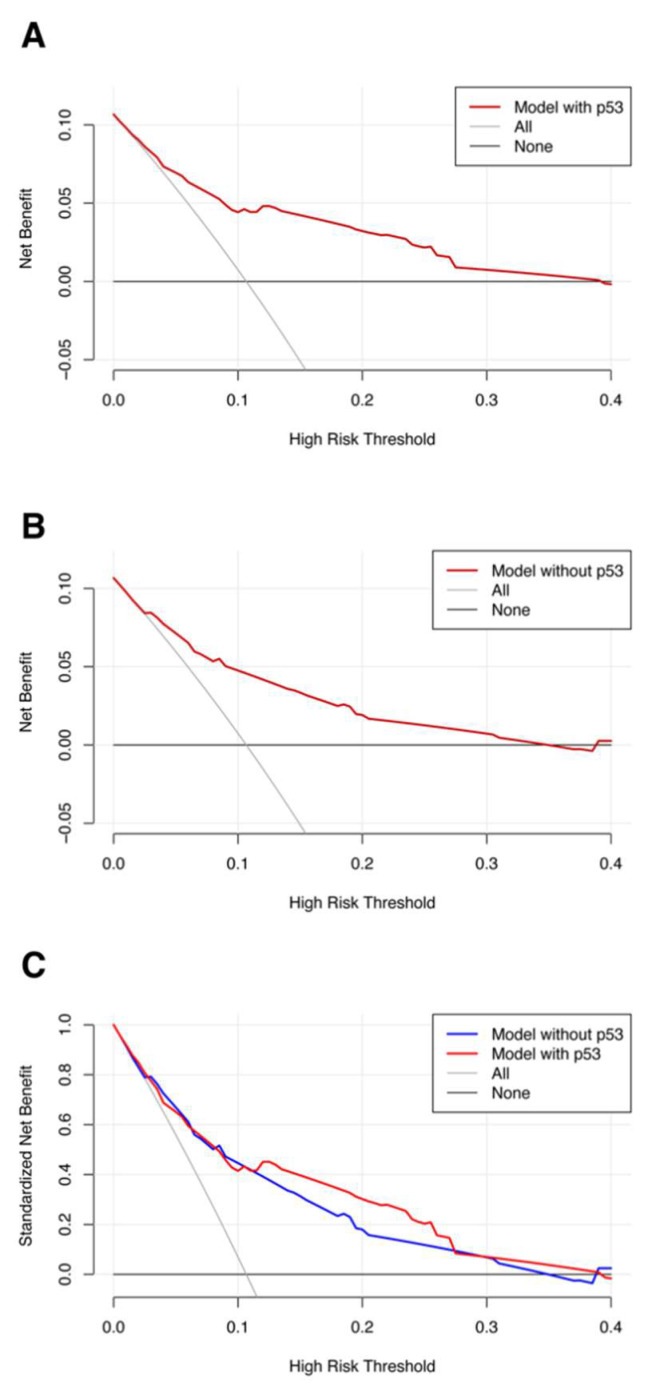
DCA of the (**A**) model with p53; (**B**) without p53. (**C**) Comparison between the models with and without p53. Abbreviations: DCA, decision curve analysis.

The DCA of the model with p53 and the model without p53 were carried out, considering it was the first pCR prediction model in BC involving the expression level of pre-treatment p53 protein with a specific value; details are shown in Figure 5. The graph shows that the net benefit of the nomogram model with p53 was higher than that without p53.

**Figure 6:**
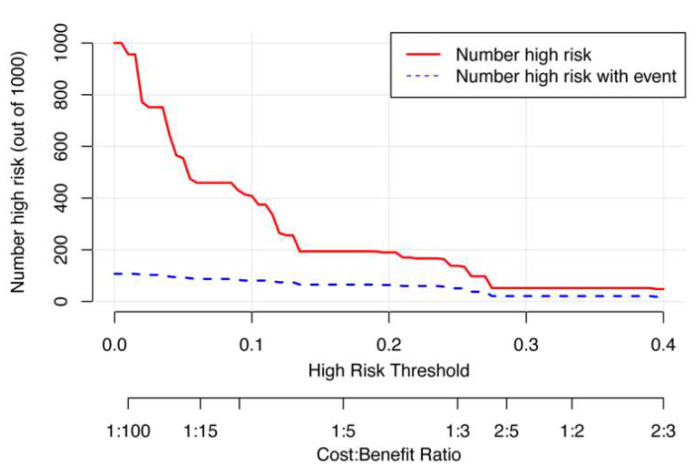
The clinical impact curves of the nomogram prediction model.

Figure 6 exhibits the clinical impact curves of the nomogram model. The blue curve (number of high risks with event) was the number of true positives at each threshold probability, while the red curve (number of high risks) represented the number of subjects classified as positive by the prediction model for each threshold probability.

## 4. Discussion

Since achieving pCR is related to better survival outcomes (event-free survival (HR = 0.40, *p* < 0.001) and overall survival (HR = 0.32, *p* < 0.001)) in the NAC setting, it has been regarded as a dependable predictor [12]. Hence, the prediction of pCR in the early pre-treatment period is of great significance. However, the predictive biomarkers in other examples in the literature are not accessible to every patient for economic reasons or the cumbersome steps involved. To fill this research gap and achieve the maximum utilization of resources, this study selected simple and easy-to-access core biopsy and clinical information of patients as predictive factors and built a prediction model for pCR in the NAC setting. It helps to predict the chemotherapy response at the time of diagnosis, and this makes it possible for clinicians to intervene early in some high-risk patients. The selection of variables and the conditions of the model development are described in the following paragraphs.

### 4.1. Clinical Tumor Staging

Clinical tumor staging plays a crucial role in chemotherapy response. Livingston-Rosanoff et al. reported a retrospective study that included 38,864 patients who underwent NAC treatment and subsequent surgery for a solitary lesion varying from cT1 to cT3, which revealed that cT3 tumors have a lower probability of achieving pCR irrespective of molecular subtypes [22], which is consistent with our study. The possible explanation for this finding is that larger tumors have a higher chance of revealing heterogeneity of elevation, which may affect the sensitivity of chemotherapy [37]. Many prediction models involved clinical tumor staging (tumor size) as a predictive factor [38,39,40], which indicates it is a reliable factor for predicting pCR.

### 4.2. Clinical Nodal Status

In the last decade, the administration of systemic treatment in patients with node-positive disease has switched from the adjuvant to the neoadjuvant setting. According to previous studies, 20–42% of firstly node-positive patients finally achieve pCR of the axillary lymph nodes [41]. Our results showed that pre-treatment clinical nodal status was associated with chemotherapy response; in other words, there was a greater possibility of pCR in patients with clinically node-negative disease, which is consistent with a previous study [21]. It reported the low probability of pathologic nodal positivity in patients with clinical node-negative and breast pCR disease [21], highlighting the crucial role of clinically node-negative in achieving pCR. Meanwhile, it indicated that clinical assessment via clinical diagnostic imaging plays a vital role in pCR prediction. Some models incorporated pre-treatment lymph node status for the prediction of axillary pCR for node-positive BC [42,43], indicating the vital role of nodal status in pCR prediction.

### 4.3. ER Status

ER, a vital factor that defines tumor subtype, has extensively been identified as a feature that affects the response to NAC [44]. Previous studies reported that ER-negative subtypes such as HER2-enriched and triple-negative BC were more likely to achieve pCR and favorable long-term outcomes [44,45], which is consistent with our results. By analyzing pre-treatment ER as a continuous variable, we could divide patients into ER-positive and ER-negative or ER-high and ER-low diseases. Further, we found the cut-off value of the pre-treatment status rather than simply dividing it into ER-positive and ER-negative, which could be explained by the fact that ER-low disease and ER-negative disease have similar biological behaviors. Weisman et al. [46] found that ER-low malignancies had a semblable pathologic response to NAC treatment as ER-negative diseases, demonstrating the above point. This will separate the ER-positive patients into different subgroups with different probabilities of achieving pCR. Nevertheless, the cut-off value remains controversial. A few previous studies assessed ER status quantitatively, one of which reported a cut-off value of 30% when distinctions in responses could be seen among patients with ER < 30% and those with ER > 30% diseases [47], which is consistent with our results showing that the cut-off value of ER was 22.5%. Additionally, the previous literature reported that the threshold of 80% best predicted the relation to pCR [48].

### 4.4. Ki67 Status

Ki-67 is a biomarker of cell proliferation used to evaluate the invasiveness of a tumor; except for the G0 phase, the expression of Ki67 exists in all the cell cycle phases [49]. Ki-67 has been assessed in several studies for its predictive role in the NAC setting, but its cut-off value remains controversial. However, a large-scale meta-analysis that incorporated 44 studies reported that high pre-treatment Ki-67 was related to elevated pCR rates in BC patients who received NAC using distinct cut-off values of Ki-67 [50]. Our study found the threshold of Ki-67 was 32.5%, which is in the range of 15% to 50%, as the previous literature reported [50].

### 4.5. p53 Status

The p53 protein, encoded by the TP53 gene, is the most frequently mutated gene in BC (especially in hormone-receptor-negative BC) [51] and plays a crucial role in metabolism, apoptosis, DNA repair, and cellular sensitivity to chemotherapy [52]. Numerous BC patients who will accept NAC treatment have cancers harboring TP53 mutations. Many studies have tried to identify the role of this mutation in pathological response, which showed that compared with wild-type counterparts, tumors with TP53 mutations have a statistically higher probability of pCR in BC [6,53]. One possible explanation is that TP53 mutations occur less frequently in luminal-type BC [51], and hormone receptor positivity is thought to be an unfavorable factor for pCR [44]. Replaced by simply dividing p53 status into positive and negative, the cut-off of 37.5% best predicted the correlation with pCR in our cohort, and patients with p53 ≥ 37.5% were more likely to have a pCR. Limited studies reported the threshold value of p53 in BC; Lee et al. [54] found that a threshold of 10% for p53 was a predictive factor of survival outcome. However, there have been no studies on the cut-off value of p53 in the NAC setting. To our knowledge, this is the first study to explore the role of pre-treatment p53 in the NAC prediction model; moreover, the cut-off predictive value of p53 was found.

### 4.6. Prediction Model

In summary, in our study, the model for predicting pCR had five variables: cT, cN, ER status, Ki67 status, and p53 status. Notably, we visualized the prediction model and presented it as a nomogram. The ROC analysis and Hosmer–Lemeshow goodness-of-fit test indicated that the model had good discrimination and calibration; AUCs of 0.804 (95% CI: 0.756–0.853) and 0.774 (95% CI: 0.649–0.899) were found for the primary and the validation cohorts, respectively; the sensitivity and specificity were 73.2% and 74.7%, respectively; Hosmer–Lemeshow goodness-of-fit test χ^2^ = 7.089, *p* = 0.527 > 0.05. In clinical applications, the probability of pCR can be judged by the total score obtained by adding the scores of each risk factor. For all we know, this is the first pCR prediction model that included the expression level of pre-treatment p53 status. The optimal model is the model with the maximum net benefit for any given probability threshold. The DCA shows that outcome prediction using the model with p53 had a greater net benefit than the model without p53. Although this is the first prediction model to involve p53 in the NAC setting, p53 has been included in other breast cancer prediction models. Meng et al. [55] developed an intraoperative model to assess the risk of non-sentinel lymph node metastasis that incorporates p53, and the AUC of the model was 0.764. On the pCR prediction model based on biopsy, Li et al. [24] developed a deep learning model with whole-slide biopsy images in the NAC setting, with an AUC of 0.72 for predicting pCR. In addition, based on the imaging, Li et al. [56] built an imaging model with diffusion-weighted MRI in neoadjuvant immunotherapy, with an AUC of 0.73 for predicting pCR. One recent study developed a prediction model with an AUC of 0.825 based on age, AJCC T stage, Ki67, HER2, and hormone receptor status [57]. However, this study had a relatively small sample size (n = 527). In contrast, the present study enrolled more patients (n = 920), and the thresholds of immunohistochemical parameters incorporating p53 were established based on ROC analysis rather than simply categorizing them as positive or negative.

Our nomogram model can be used to predict the probability of pCR for each patient, thus helping clinicians to make better decisions. Recent studies have focused on individualized treatment, increasing and de-escalating treatment for non-responders and large responders, respectively [14,15]. Our nomogram model may be useful for clinical studies to select patients for de-escalating treatment. In addition, performing pCR predictions may create opportunities for patients who have considerable residual disease after conventional NAC treatment to become candidates for clinical trials of innovative drugs. For those patients with tumor residuals after NAC, the systematic management of post-NAC therapy is also required [58]. The better selection of patients for more appropriate treatment based on biomarkers, both in NAC and in post-NAC treatment, is an Issue that will need to be explored over time.

Trastuzumab was included in Chinese health insurance in 2017, and our primary cohort included patients who underwent treatment in 2017 and earlier. Due to health insurance and economic factors, only an extremely small number of HER2-positive patients received neoadjuvant targeted therapy, and a higher proportion of hormone-receptor-positive patients were included, and these reasons may have contributed to the lower pCR rates than other studies that combined NAC with targeted therapy or included a lower proportion of hormone-receptor-positive patients [59]. This study examined the predictive factors of NAC per se for pCR in breast cancer. In recent years, with the development of therapeutic agents and advances in breast cancer treatment strategies, more and more treatment options are being used in neoadjuvant therapy, such as neoadjuvant targeted therapy, neoadjuvant endocrine therapy, and neoadjuvant immunotherapy [60], and more studies are needed to explore the role of these options, alone and in combination, in neoadjuvant therapy.

## 5. Strengths and Limitations

In contrast to prior studies [24,55,57], the ER, PR, Ki67, and p53 thresholds were established based on ROC analysis rather than simply categorizing them as positive or negative. In order to facilitate clinical application, we created a user-friendly nomogram based on our Cox model. Compared to previous models [25,56], the predictors in our model are included in routine domestic tests with standardized test specifications and no additional economic costs. An additional benefit of this study is the utilization of a sizable sample size, which increases the process’s reproducibility and comparability.

The major limitation of this study is that the prediction model was developed based on the Chinese population and may not apply to other ethnic groups. In addition, external validation was carried out at the same center, and it is hoped that multi-center validation will be carried out in further studies. Furthermore, having a larger sample size and training predictive models for each cancer subtype would be helpful in clinical practice.

## 6. Conclusions

The model developed in this study incorporated five variables, cT, cN, ER status, Ki67 status, and p53 status, which had good predictive power for pCR. The model is simple, and the selected predictors are all easy-to-access variables from core biopsy and clinical information, which makes it easy to utilize and can be widely applied to BC patients who received NAC to identify groups that need early intervention.

## Figures and Tables

**Table 1:** Baseline clinicopathological characteristics in the primary cohort and univariate analysis.

Predictive Factors	Comparison of Predictive Factors between the Two Groups [M (P25, P75)]				
	All Subjects (*n* = 769) *n* (%)	pCR Group (*n* = 82) *n* (%)	Non-pCR Group (*n* = 687) *n* (%)	Z/χ^2^	*p*
Age at diagnosis, y	48.0 (43.0–56.0)	47.0 (41.8–54.0)	48.0 (43.0–56.0)	−1.225	0.221
Menopausal status				0.069	0.812
Pre-menopause	468 (60.9%)	51 (62.2%)	417 (60.7%)		
Post-menopause	301 (39.1%)	31 (37.8%)	270 (39.3%)		
cT				16.117	<0.001
cT1	48 (6.2%)	13 (15.9%)	35 (5.1%)		
cT2	537 (69.8%)	56 (68.3%)	481 (70.0%)		
cT3 + cT4	184 (23.9%)	13 (15.9%)	171 (24.9%)		
cN				26.898	<0.001
Negative	303 (39.4%)	54 (65.9%)	249 (36.2%)		
Positive	466 (60.6%)	28 (34.1%)	438 (63.8%)		
ER status (%)				30.283	<0.001
<22.5	361 (46.9%)	62 (75.6%)	299 (43.5%)		
≥22.5	408 (53.1%)	20 (24.4%)	388 (56.5%)		
PR status (%)				17.836	<0.001
<6.5	432 (56.2%)	64 (78.0%)	368 (53.6%)		
≥6.5	337 (43.8%)	18 (22.0%)	319 (46.4%)		
HER2 status				0.376	0.557
Negative	437 (56.8%)	44 (53.7%)	393 (57.2%)		
Positive	332 (43.2%)	38 (46.3%)	294 (42.8%)		
Ki67 status (%)				23.974	<0.001
<32.5	552 (71.8%)	40 (48.8%)	512 (74.5%)		
≥32.5	217 (28.2%)	42 (51.2%)	175 (25.5%)		
p53 status (%)				20.847	<0.001
<37.5	426 (55.4%)	26 (31.7%)	400 (58.2%)		
≥37.5	343 (44.6%)	56 (68.3%)	287 (41.8%)		
Chemotherapy cycles				0.247	0.619
4	705 (91.7%)	74 (90.2%)	631 (91.8%)		
5–8	64 (8.3%)	8 (9.8%)	56 (8.2%)		

pCR, pathological complete response; cT, clinical T staging; cN, clinical nodal status; ER, estrogen receptor; PR, progesterone receptor; HER2, human epidermal growth factor receptor 2.

**Table 2:** Multicollinearity test of predictive factors and the approach to assigning values.

Factors	Tolerance	VIF	Assignment
cT cN ER status PR status Ki67 status p53 status	0.986 0.964 0.489 0.499 0.938 0.948	1.014 1.037 2.046 2.004 1.067 1.054	“cT1” = 1, “cT2” = 2, “cT3” = 3 “Negative” = 0, “Positive” = 1 “<22.5%” = 0, “≥22.5%” = 1 “<6.5%” = 0, “≥6.5%” = 1 “<32.5%” = 0, “≥32.5%” = 1 “<37.5%” = 0, “≥37.5%” = 1

VIF, variance inflation factor; cT, clinical T staging; cN, clinical nodal status; ER, estrogen receptor; PR, progesterone receptor.

**Table 3:** Results of binary logistic regression analysis.

Predictive Factors	B	SE	Wals	*p*	OR (95% CI)
cT					
cT1					Ref.
cT2 cT3 + cT4 cN: neg vs. pos ER status: <22.5% vs. ≥22.5% Ki67 status: <32.5% vs. ≥32.5% p53 status: <37.5% vs. ≥37.5% Constants	−1.550 −1.963 1.045 1.237 −0.851 −0.917 −0.994	0.403 0.483 0.262 0.288 0.258 0.267 0.466	14.802 16.526 15.900 18.498 10.861 11.847 4.541	<0.001 <0.001 <0.001 <0.001 0.001 0.001 0.033	0.212 (0.096–0.468) 0.140 (0.054–0.362) 2.843 (1.701–4.751) 3.446 (1.961–6.056) 0.427 (0.257–0.708) 0.400 (0.237–0.674) 0.370

SE, standard error; OR, odds ratio; CI, confidence interval; cT, clinical T staging; cN, clinical nodal status; ER, estrogen receptor.

## Data Availability

The original datasets used and/or analyzed during the present study are available from the corresponding author upon reasonable request.

## Supplementary Materials

The following supporting information can be downloaded at: https://www.mdpi.com/article/10.3390/ijerph20021617/s1, Table S1. Clinical characteristics of patients in the primary and validation cohorts. Table S2. Relationship between cancer subtype and pathological complete response (N, %).
