# Supplementary material for: Development and Assessment of a Novel Core Biopsy-Based Prediction Model for Pathological Complete Response to Neoadjuvant Chemotherapy in Women with Breast Cancer
Source: Int J Environ Res Public Health. 2023 Jan 16;20(2):1617. doi: 10.3390/ijerph20021617 (PMC9867383; doi:10.3390/ijerph20021617)
Supplement: Supplementary file 1 [file ijerph-20-01617-s001.zip › ijerph-2081896-supplementary.pdf]

Supplementary Table S1. Clinical characteristics of patients in the primary and validation cohorts.

Predictive factors	Primary cohort (n=769) n (%)	Validation cohort (n=151) n (%)	P
Age at diagnosis, y	48.0 (43.0-56.0)	49.0 (44.0-56.0)	0.265
Menopausal status			0.927
Pre-menopause	468 (60.9%)	91 (60.3%)	
Post-menopause	301 (39.1%)	60 (39.7%)	
cT			0.549
cT1	48 (6.2%)	7 (4.6%)	
cT2	537 (69.8%)	112 (74.2%)	
cT3+cT4	184 (23.9%)	32 (21.2%)	
cN			0.120
Negative	303 (39.4%)	49 (32.5%)	
Positive	466 (60.6%)	102 (67.5%)	
ER status (%)			0.327
<22.5	361 (46.9%)	64 (42.4%)	
≥22.5	408 (53.1%)	87 (57.6%)	
PR status (%)			0.929
<6.5	432 (56.2%)	86 (57.0%)	
≥6.5	337 (43.8%)	65 (43.0%)	
HER2 status			0.591
Negative	437 (56.8%)	82 (54.3%)	
Positive	332 (43.2%)	69 (45.7%)	
Ki67 status (%)			0.051
<32.5	552 (71.8%)	96 (63.6%)	
≥32.5	217 (28.2%)	55 (36.4%)	
p53 status (%)			0.370
<37.5	426 (55.4%)	90 (59.6%)	
≥37.5	343 (44.6%)	61 (40.4%)	
Chemotherapy cycles			0.091
4	705 (91.7%)	145 (96.0%)	
5-8	64 (8.3%)	6 (4.0%)	

cT clinical T staging, cN clinical nodal status, ER estrogen receptor, PR progesterone receptor, HER2 human epidermal growth factor receptor 2.

Supplementary Table S2. Relationship between cancer subtype and pathological complete response (N, %).

Cancer subtype	Primary cohort (n=769) n (%)			Validation cohort (n=151) n (%)		
	Non-pCR	pCR	Total	Non-pCR	pCR	Total
HR(+), HER2(-)	292 (93.6%)	20 (6.4%)	312 (40.6%)	59 (95.2%)	3 (4.8%)	62 (41.1%)
HR(+), HER2(+)	157 (91.3%)	15 (8.7%)	172 (22.4%)	33 (91.7%)	3 (8.3%)	36 (23.8%)
HR(-), HER2(+)	137 (85.6%)	23 (14.4%)	160 (20.8%)	27 (81.8%)	6 (18.2%)	33 (21.9%)
HR(-), HER2(-)	101 (80.8%)	24 (19.2%)	125 (16.3%)	15 (75.0%)	5 (25.0%)	20 (13.2%)

pCR pathological complete response, HR hormone receptor, HER2 human epidermal growth factor receptor 2.
